# Supplementary material for: Assessing Uncertainty in the Rooting of the SARS-CoV-2 Phylogeny
Source: Mol Biol Evol. 2020 Dec 9;38(4):1537–43. doi: 10.1093/molbev/msaa316 (PMC7798932; doi:10.1093/molbev/msaa316)
Supplement: msaa316_Supplementary_Data [file msaa316_supplementary_data.zip › rootMS_Supplement.pdf]

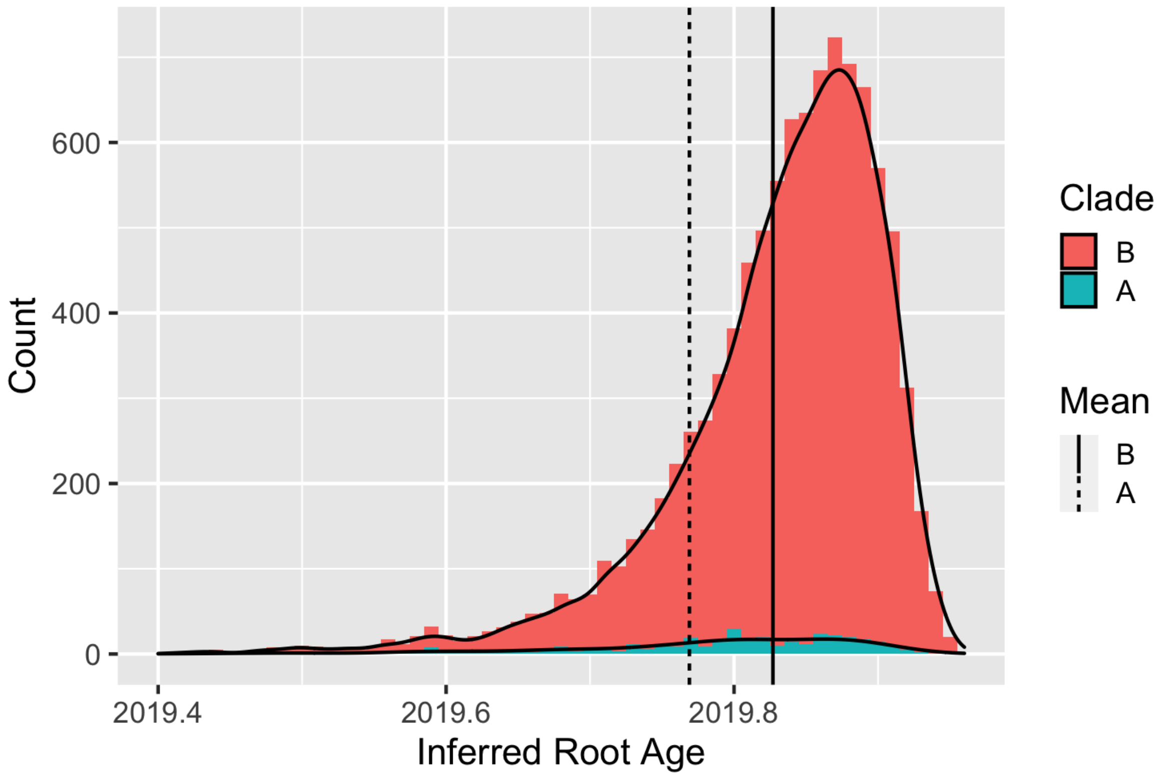

FIG. S1. Distributions of inferred root ages for 10,000 BEAST sampled trees with roots in clade A vs. clade B. 9,616 sampled trees have roots in clade B while 384 sampled trees have roots in clade A. Parameters for the BEAST analysis are described in the caption for Figure 1. Vertical lines represent the means in the two distributions. The mean in the clade A distribution is 2019.769 and the mean in the clade B distribution is 2019.827.

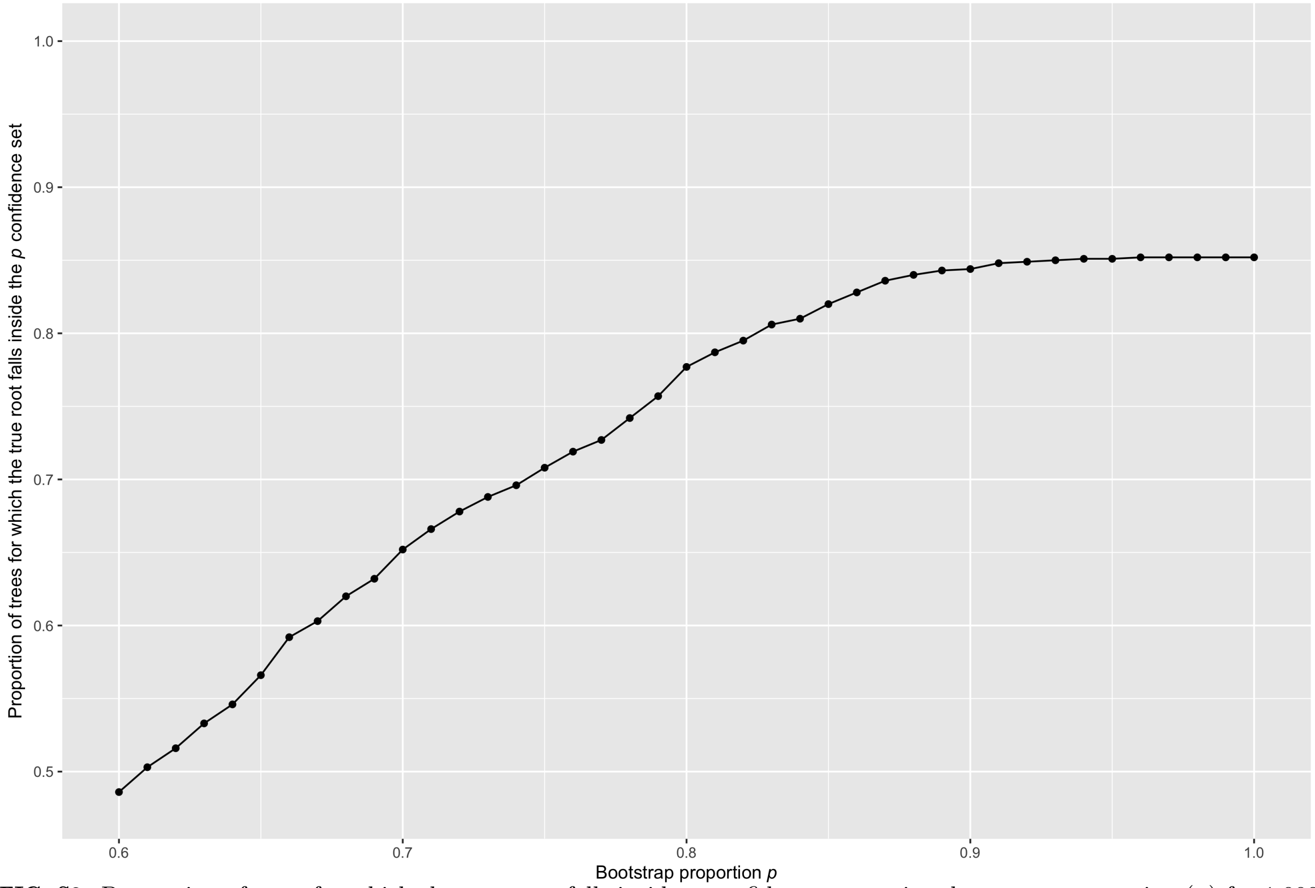

FIG. S2. Proportion of trees for which the true root falls inside p confidence set against bootstrap proportion (p) for 1,000 parametric simulations. Simulations were performed with pyvolve (Spielman and Wilke, 2015) using maximum likelihood estimates, from the original data set, of the model of molecular evolution and the phylogenetic tree, including branch lengths (see Table S2).

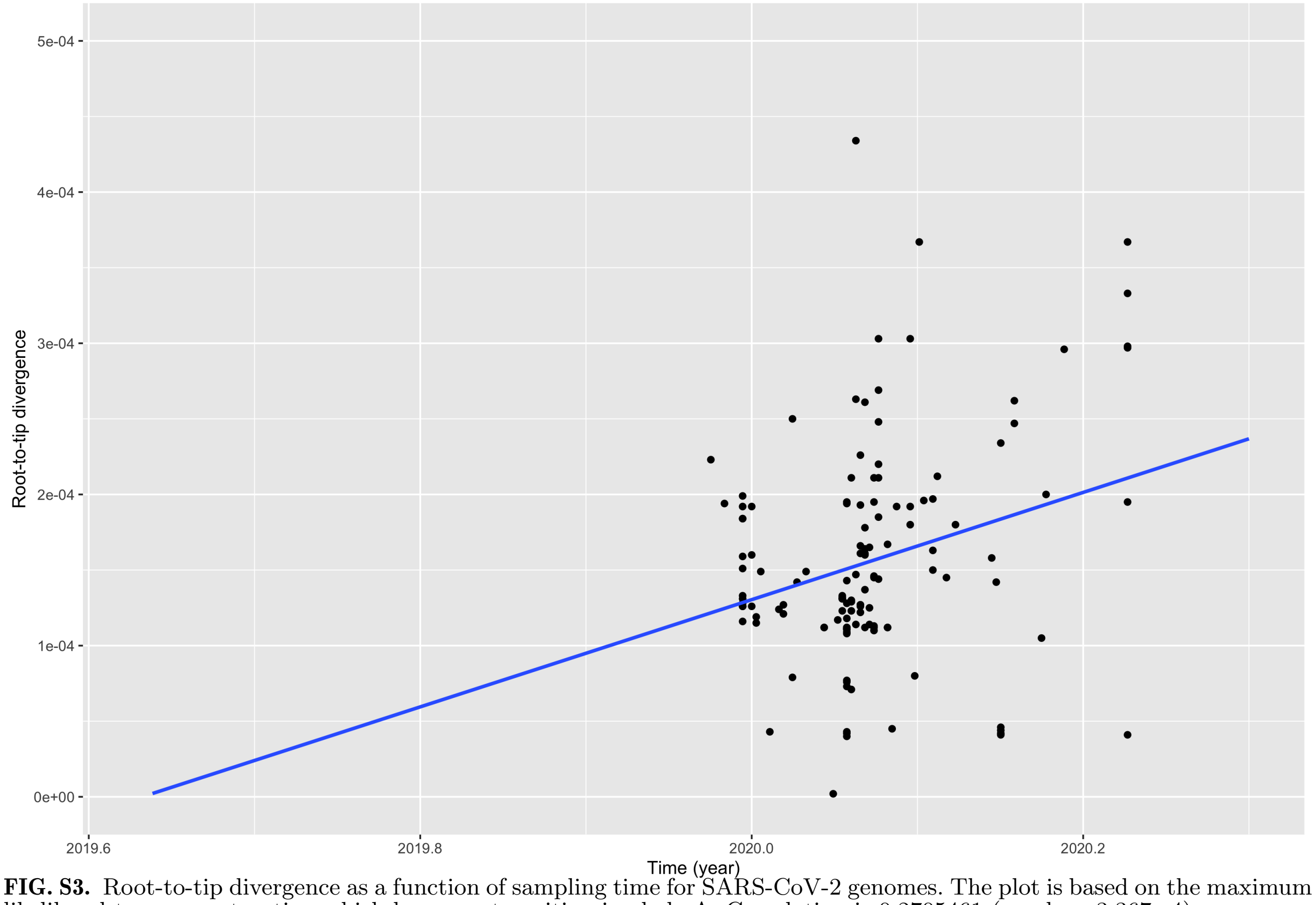

FIG. S3. Root-to-tip divergence as a function of sampling time for SARS-CoV-2 genomes. The plot is based on the maximum likelihood tree reconstruction which has a root position in clade A. Correlation is 0.2705461 (p-value=3.367e-4).

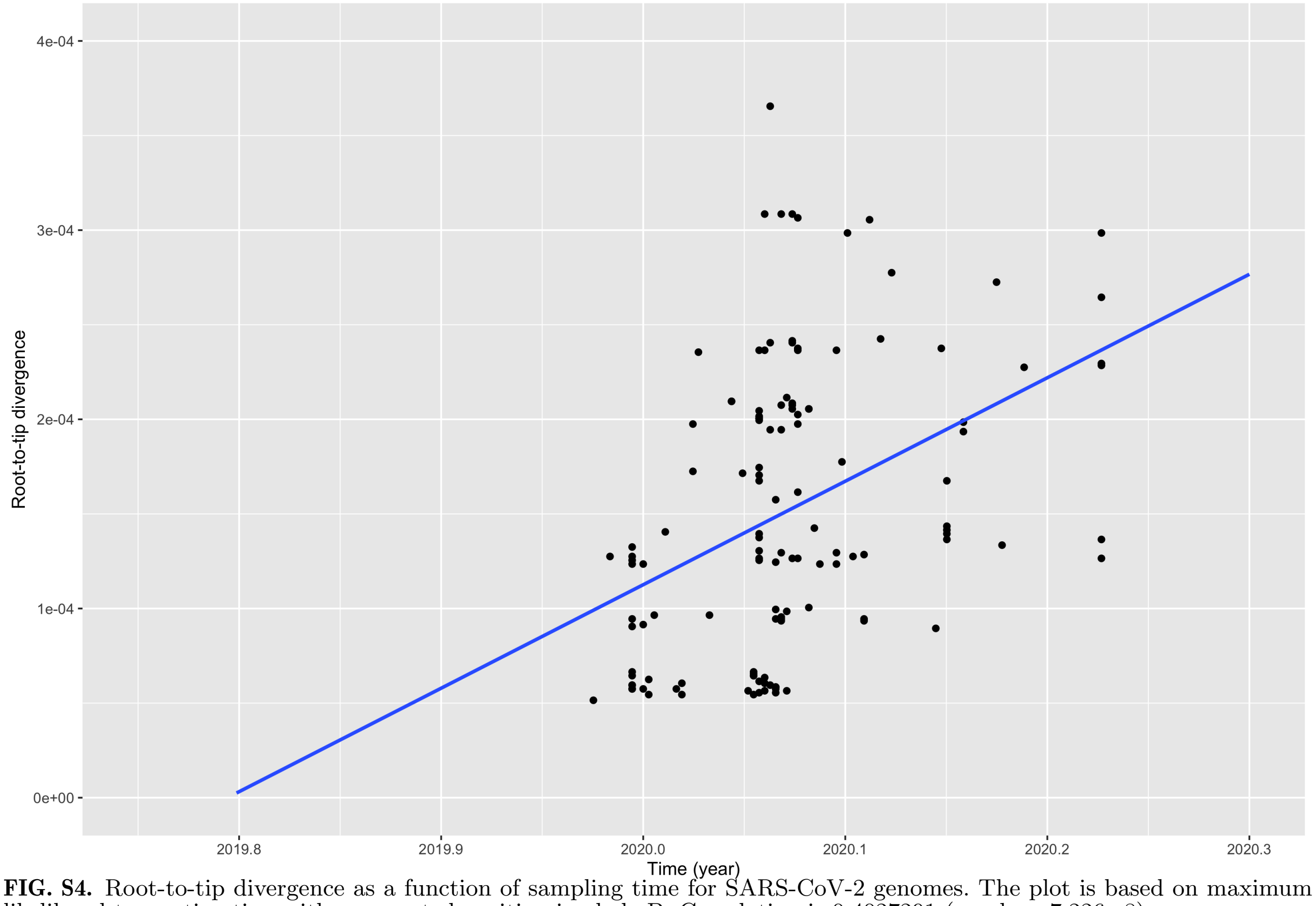

FIG. S4. Root-to-tip divergence as a function of sampling time for SARS-CoV-2 genomes. The plot is based on maximum likelihood tree estimation with a re-rooted position in clade B. Correlation is 0.4027291 (p-value=7.226e-8).

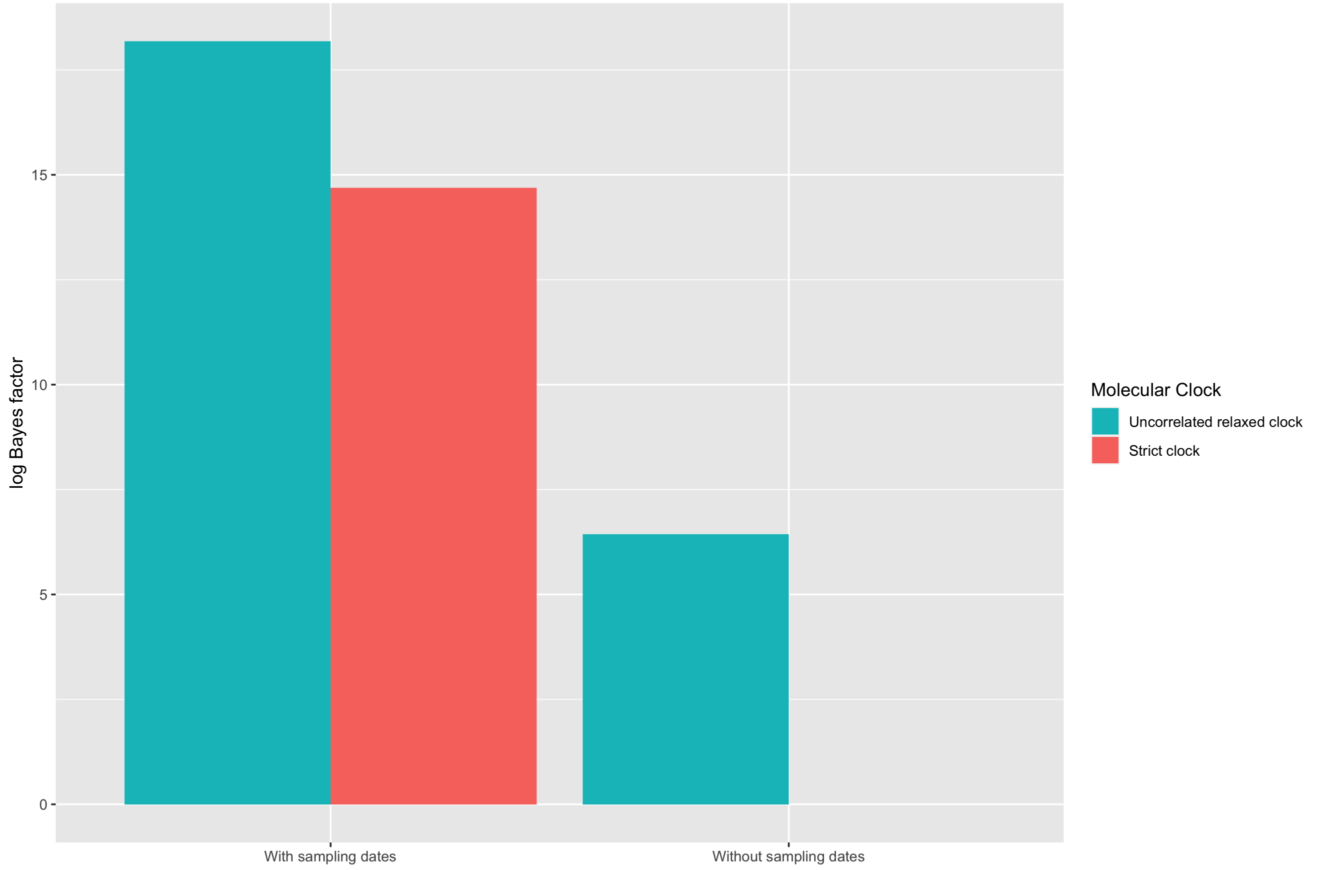

FIG. S5. Bayesian evaluation of temporal signal (BETS) results where the poorest performing model has a log Bayes factor of 0. We analyzed the sequences in Table S1 using BEAST 1.10.4 using a MCMC length of 10,000,000 steps and sampling every 1,000 steps. We compared the statistical fit of two molecular clock models, strict clock and uncorrelated lognormal relaxed clock, with and without sampling times. For each combination of molecular clock and sampling times, we calculated the (log) marginal likelihood using generalized stepping stone sampling (Baele *et al.*, 2016), for which we used 200 path steps with a chain length for each 100000 iterations.

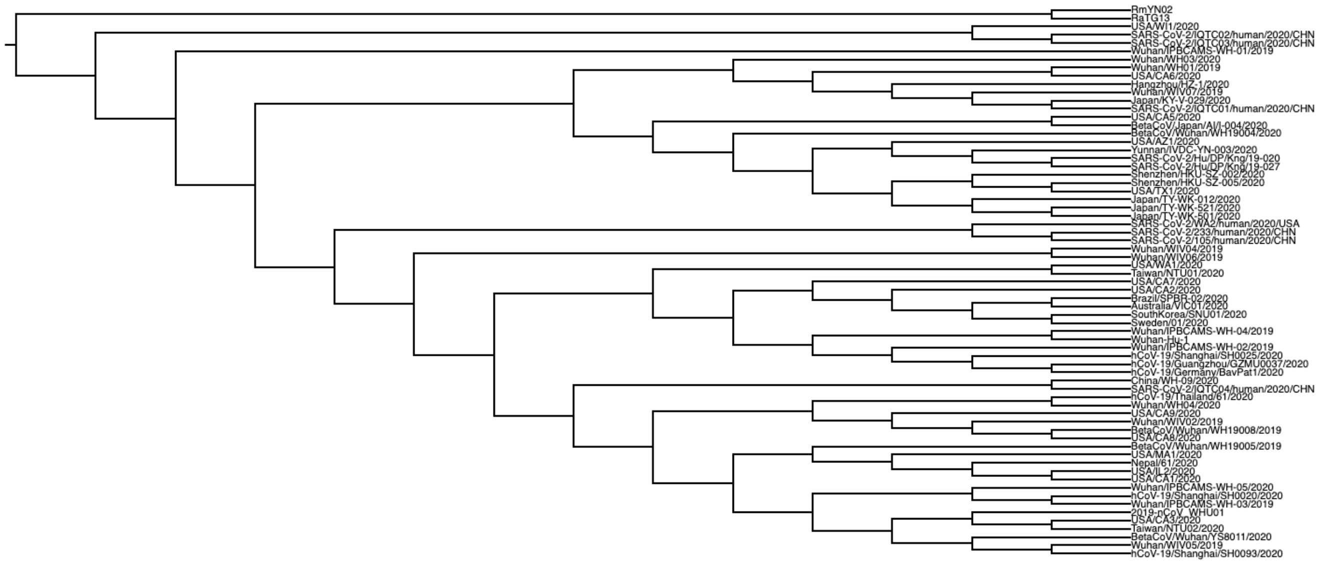

FIG. S6. Outgroup rooting by removal of positions 8782, 18060, and 28144 from the alignment.

Table S1.: Accessions and sources of the SARS-CoV-2 genome sequences used in this study.

Genome Name	Source	Accession ID
Wuhan/IPBCAMS-WH-01/2019	GenBank	MT019529
BetaCoV/Wuhan/WH-01/2019	GenBank	LR757998
Wuhan/WIV02/2019	GenBank	MN996527
Wuhan/WIV04/2019	GenBank	MN996528
Wuhan/WIV05/2019	GenBank	MN996529
Wuhan/WIV06/2019	GenBank	MN996530
Wuhan/WIV07/2019	GenBank	MN996531
Wuhan/IPBCAMS-WH-02/2019	GenBank	MT019530
Wuhan/IPBCAMS-WH-03/2019	GenBank	MT019531
Wuhan/IPBCAMS-WH-04/2019	GenBank	MT019532
BetaCoV/Wuhan/WH19008/2019	NMDC	NMDC60013002_06
BetaCoV/Wuhan/WH19005/2019	NMDC	NMDC60013002_10
2019-nCoV_HKU-SZ-002a_2020	GenBank	MN938384
2019-nCoV_HKU-SZ-005b_2020	GenBank	MN975262
SouthKorea/SNU01/2020	GenBank	MT039890
BetaCoV/Wuhan/WH-03/2019	GenBank	LR757996
Wuhan/IPBCAMS-WH-05/2020	GenBank	MT019533
BetaCoV/Wuhan/WH19004/2020	NMDC	NMDC60013002_09
2019-nCoV_WHU01	GenBank	MN988668
Wuhan/WH04/2019	GenBank	LR757995
BetaCoV/Wuhan/YS8011/2020	NMDC	NMDC60013002_07
China/WH-09/2020	GenBank	MT093631
Nepal/61/2020	GenBank	MT072688
SARS-CoV-2/human/CHN/Yunnan-01/2020	GenBank	MT049951
USA/WA1/2020	GenBank	MN985325
Hangzhou/HZ-1/2020	GenBank	MT039873
USA/CA2/2020	GenBank	MN994468

USA-AZ1/2020	GenBank	MN997409
USA/CA1/2020	GenBank	MN994467
BetaCoV/Japan/AI/I-004/2020	GenBank	LC521925
Australia/VIC01/2020	GenBank	MT007544
SARS-CoV-2/105/human/2020/CHN	GenBank	MT135041
USA/CA6/2020	GenBank	MT044258
USA/IL2/2020	GenBank	MT044257
SARS-CoV-2/233/human/2020/CHN	GenBank	MT135043
Japan/KY/V-029/2020	GenBank	LC522972
Japan/TY/WK-012/2020	GenBank	LC522973
USA/CA3/2020	GenBank	MT027062
USA/CA5/2020	GenBank	MT027064
SARS-CoV-2/IQTC02/human/2020/CHN	GenBank	MT123291
Japan/TY/WK-501/2020	GenBank	LC522974
Japan/TY/WK-521/2020	GenBank	LC522975
USA/WI1/2020	GenBank	MT039887
Taiwan/NTU01/2020	GenBank	MT066175
Taiwan/NTU02/2020	GenBank	MT066176
SARS-CoV-2/IQTC01/human/2020/CHN	GenBank	MT123290
USA/CA7/2020	GenBank	MT106052
Sweden/01/2020	GenBank	MT093571
SARS-CoV-2/Hu/DP/Kng/19-020	GenBank	LC528232
SARS-CoV-2/Hu/DP/Kng/19-027	GenBank	LC528233
USA/CA8/2020	GenBank	MT106053
USA/CA9/2020	GenBank	MT118835
SARS-CoV-2/IQTC04/human/2020/CHN	GenBank	MT123292
SARS-CoV-2/IQTC03/human/2020/CHN	GenBank	MT123293
Brazil/SPBR-02/2020	GenBank	MT126808
SARS-CoV-2/WA2/human/2020/USA	GenBank	MT152824
Wuhan-Hu-1	GenBank	NC_045512

hCoV-19/Thailand/61/2020	GISAID	EPI_ISL_403962
hCoV-19/Germany/BavPat1/2020	GISAID	EPI_ISL_406862
hCoV-19/Guangzhou/GZMU0037/2020	GISAID	EPI_ISL_416334
hCoV-19/Shanghai/SH0025/2020	GISAID	EPI_ISL_416334
hCoV-19/Zhejiang/HZ103/2020	GISAID	EPI_ISL_422425
SARS-CoV-2/human/CHN/Beijing_IME-BJ01/2020	GenBank	MT291831.1
USA/TX1/2020	GenBank	MT106054
SARS-CoV-2/human/CHN/WHUHnCoV003/2020	GenBank	MT079845
SARS-CoV-2/human/CHN/CN2/2020	GenBank	MT407650
SARS-CoV-2/human/CHN/CN1/2020	GenBank	MT407649
SARS-CoV-2/human/CHN/CN5/2020	GenBank	MT407651
SARS-CoV-2/IQTC01/human/2020/CHN	GenBank	MT123290
SARS-CoV-2/human/CHN/HZ-477/2020	GenBank	MT253699
SARS-CoV-2/human/CHN/HZ-178/2020	GenBank	MT253697
SARS-CoV-2/human/CHN/HZ-62/2020	GenBank	MT253706
SARS-CoV-2/human/CHN/HZ-90/2020	GenBank	MT253709
SARS-CoV-2/human/CHN/HZ-48/2020	GenBank	MT253701
SARS-CoV-2/human/CHN/Wuhan_IME-WH05/2019	GenBank	MT291830
SARS-CoV-2/human/CHN/HZ-49/2020	GenBank	MT253702
SARS-CoV-2/human/CHN/HZ-185/2020	GenBank	MT253698
SARS-CoV-2/human/CHN/HZ-79/2020	GenBank	MT253708
SARS-CoV-2/human/CHN/GZMU0014/2020	GenBank	MT568634
SARS-CoV-2/human/CHN/YN-0306-466/2020	GenBank	MT396241
SARS-CoV-2/human/CHN/Beijing_IME-BJ05/2020	GenBank	MT291835
SARS-CoV-2/human/Guangzhou/IQTC05/2020	GenBank	MT446312
SARS-CoV-2/human/CHN/Wuhan$_{Y}B$012506/2020	GenBank	MT259230
Wuhan/WH01/2019	GenBank	MT291826
SARS-CoV-2/human/CHN/Wuhan_YB012602/2020	GenBank	MT259229
SARS-CoV-2/human/CHN/Wuhan_YB012504/2020	GenBank	MT259231
SARS-CoV-2/human/CHN/Wuhan_YB012605/2020	GenBank	MT259228

SARS-CoV-2/human/CHN/Wuhan_YB012611/2020	GenBank	MT259227
SARS-CoV-2/human/CHN/HZ-481/2020	GenBank	MT253700
SARS-CoV-2/human/CHN/HZ-91/2020	GenBank	MT253710
Wuhan/WH03/2020	GenBank	MT291828
SARS-CoV-2/human/CHN/HZ-576/2020	GenBank	MT253704
SARS-CoV-2/human/CHN/HZ-638/2020	GenBank	MT253707
SARS-CoV-2/human/CHN/HZ-551/2020	GenBank	MT253703
SARS-CoV-2/human/CHN/WHUHnCoV021/2020	GenBank	MT079854
SARS-CoV-2/human/CHN/SH01/2020	GenBank	MT121215
SARS-CoV-2/human/CHN/HZ-162/2020	GenBank	MT253696
SARS-CoV-2/human/CHN/Changzhou_JS27/2020	GenBank	MT534630
hCoV-19/Germany/BavPat1/2020	GenBank	MT270101
SARS-CoV-2/human/CHN/OS6/2020	GenBank	MT407656
SARS-CoV-2/human/CHN/OS3/2020	GenBank	MT407657
SARS-CoV-2/human/CHN/OS4/2020	GenBank	MT407659
SARS-CoV-2/human/CHN/Fuyang_FY002/2020	GenBank	MT281577
SARS-CoV-2/human/CHN/OS5/2020	GenBank	MT407655
SARS-CoV-2/human/CHN/OS1/2020	GenBank	MT407654
SARS-CoV-2/human/CHN/HZ-60/2020	GenBank	MT253705
SARS-CoV-2/human/CHN/Wuhan_OS52/2020	GenBank	MT259226
2019-nCoV_WHU02	GenBank	MN988669
SARS-CoV-2/human/CHN/Beijing-01/2020	GenBank	MT034054
SARS-CoV-2/human/CHN/CN3/2020	GenBank	MT407652
SARS-CoV-2/human/CHN/Shanghai_CH-02/2020	GenBank	MT627325
SARS-CoV-2/human/CHN/WHUHnCoV011/2020	GenBank	MT079851
SARS-CoV-2/human/CHN/WHUHnCoV001/2020	GenBank	MT079843
SARS-CoV-2/human/CHN/WHUHnCoV004/2020	GenBank	MT079846
SARS-CoV-2/human/CHN/WHUHnCoV020/2020	GenBank	MT079853
SARS-CoV-2/human/CHN/WHUHnCoV002/2020	GenBank	MT079844
SARS-CoV-2/human/CHN/WHUHnCoV008/2020	GenBank	MT079850

SARS-CoV-2/human/CHN/GZMU0047/2020	GenBank	MT568640
SARS-CoV-2/human/CHN/OS2/2020	GenBank	MT407658
SARS-CoV-2/human/CHN/Beijing_IME-BJ03/2020	GenBank	MT291833
SARS-CoV-2/human/CHN/231/2020	GenBank	MT135042
SARS-CoV-2/human/CHN/235/2020	GenBank	MT135044
SARS-CoV-2/human/CHN/Beijing_IME-BJ04/2020	GenBank	MT291834
SARS-CoV-2/human/CHN/WHUHnCoV005/2020	GenBank	MT079847
SARS-CoV-2/human/CHN/WHUHnCoV006/2020	GenBank	MT079848
Yunnan/IVDC-YN-003/2020	GISAID	EPI_ISL_408480
SARS-CoV-2/human/CHN/WHUHnCoV007/2020	GenBank	MT079849
SARS-CoV-2/human/CHN/GZMU0044/2020	GenBank	MT568639
SARS-CoV-2/human/CHN/WHUHnCoV007/2020	GenBank	MT079849
SARS-CoV-2/human/CHN/GZMU0044/2020	GenBank	MT568639
SARS-CoV-2/human/CHN/GZMU0048/2020	GenBank	MT568641
Meizhou_MZ02/2020	GenBank	MT510727
Meizhou_MZ01/2020	GenBank	MT510728
SARS-CoV-2/human/CHN/GZMU0016/2020	GenBank	MT568635
SARS-CoV-2/human/CHN/WHUHnCoV012/2020	GenBank	MT079852
SARS-CoV-2/human/CHN/Shanghai_CH-03/2020	GenBank	MT622319
SARS-CoV-2/human/CHN/CN4/2020	GenBank	MT407653
RaTG13	GenBank	MN996532.1
RmYN02	GISAID	EPI_ISL_412977

Table S2. Nucleotide frequencies and mutation rates used in the parametric simulations.

Nucleotide	A	G	C	T
Frequency	0.298101	0.183265	0.195801	0.322833

Mutations	A→C	A→G	A→T	C→G	C→T	G→T
Rate	0.767496	5.729515	1.093530	0.575406	16.142857	1

((((((((((((SARS-CoV-2/human/CHN/Beijing$_I$ME−BJ01/2020:0.000069,Shenzhen/HKU−SZ−
002/2020:0.000001):0.000001,USA/AZ1/2020:0.000034):0.000001,(Japan/TY−WK−012/2020:
0.000034,(Japan/TY−WK−501/2020:0.000001,Japan/TY−WK−521/2020:0.000001):0.000001):
0.000034):0.000001,USA/TX1/2020:0.000137):0.000001,Shenzhen/HKU−SZ−005/2020:0.000068):
0.000034,SARS−CoV−2/human/CHN/WHUHnCoV003/2020:0.000103):0.000001,(((SARS−
CoV−2/human/CHN/CN2/2020:0.000034,SARS−CoV−2/human/CHN/CN1/2020:
0.000001):0.000001,SARS−CoV−2/human/CHN/CN5/2020:0.000034):0.000034,(((((SARS−
CoV−2/IQTC01/human/2020/CHN:0.000001,Japan/KY−V−029/2020:0.000069):
0.000068,((SARS−CoV−2/human/CHN/HZ−477/2020:0.000001,(((((((((SARS−CoV−
2/human/CHN/HZ−178/2020:0.000001,(BetaCoV/Wuhan/YS8011/2020:0.000001,((((SARS−
CoV−2/human/CHN/HZ−62/2020:0.000001,(((SARS−CoV−2/human/CHN/HZ−90/2020:
0.000001,(SARS−CoV−2/human/CHN/HZ−48/2020:0.000001,Wuhan/WIV05/2019:
0.000068):0.000001):0.000001,(SARS−CoV−2/human/CHN/Wuhan$_I$ME−WH05/2019:
0.000001,SARS−CoV−2/human/CHN/HZ−49/2020:0.000001):0.000001):0.000001,(SARS−
CoV−2/human/CHN/HZ−185/2020:0.000001,(((Wuhan/IPBCAMS−WH−02/2019:
0.000001,SARS−CoV−2/human/CHN/HZ−79/2020:0.000001):0.000001,(SARS−CoV−
2/human/CHN/GZMU0014/2020:0.000102,SARS−CoV−2/human/CHN/YN−0306−
466/2020:0.000068):0.000001):0.000001,(SARS−CoV−2/human/CHN/Beijing$_I$ME−
BJ05/2020:0.000034,(((SARS−CoV−2/IQTC02/human/2020/CHN:0.000034,(SARS−CoV−
2/IQTC03/human/2020/CHN:0.000034,SARS−CoV−2/human/Guangzhou/IQTC05/2020:
0.000034):0.000034):0.000069,USA/WI1/2020:0.000001):0.000001,SARS−CoV−
2/human/CHN/Wuhan$_Y$B012506/2020:0.000001):0.000034):0.000001):0.000001):0.000001):
0.000001):0.000001,hCoV−19/Thailand/61/2020:0.000001):0.000001,Wuhan/WH01/2019:
0.000069):0.000001,((SARS−CoV−2/human/CHN/Wuhan$_Y$B012602/2020:
0.000001,(SARS−CoV−2/human/CHN/Wuhan$_Y$B012504/2020:0.000001,SARS−CoV−

2/human/CHN/Wuhan$_Y$B012605/2020:0.000001):0.000001):0.000001,SARS−CoV−
2/human/CHN/Wuhan$_Y$B012611/2020:0.000103):0.000034):0.000001):0.000001):0.000001,SARS−
CoV−2/human/CHN/HZ−481/2020:0.000001):0.000001,China/WH−09/2020:0.000001):
0.000001,(((SARS−CoV−2/human/CHN/HZ−91/2020:0.000001,(((Wuhan/WH03/2020:
0.000001,SARS−CoV−2/human/CHN/HZ−576/2020:0.000001):0.000001,Taiwan/NTU02/2020:
0.000068):0.000001,(BetaCoV/Wuhan/WH19005/2019:0.000068,((SARS−CoV−
2/human/CHN/HZ−638/2020:0.000001,SARS−CoV−2/human/CHN/HZ−
551/2020:0.000001):0.000001,(SARS−CoV−2/human/CHN/WHUHnCoV021/2020:
0.000068,Wuhan/IPBCAMS−WH−05/2020:0.000034):0.000001):0.000001):0.000001):
0.000001):0.000001,(((BetaCoV/Japan/AI/I−004/2020:0.000069,SARS−CoV−
2/human/CHN/SH01/2020:0.000068):0.000001,((Wuhan/IPBCAMS−WH−04/2019:
0.000001,Wuhan/WIV06/2019:0.000001):0.000001,USA/CA9/2020:0.000034):0.000001):
0.000001,((((((SARS−CoV−2/human/CHN/HZ−162/2020:0.000001,((SARS−CoV−
2/human/CHN/Changzhou$_J$S27/2020:0.000001,SARS−CoV−2/Hu/DP/Kng/19−
027:0.000034):0.000001,SARS−CoV−2/Hu/DP/Kng/19−020:0.000001):0.000034):
0.000001,Wuhan/WIV04/2019:0.000001):0.000001,(hCoV−19/Germany/BavPat1/2020:
0.000001,(hCoV−19/Guangzhou/GZMU0037/2020:0.000001,(((SARS−CoV−
2/human/CHN/OS6/2020:0.000069,SARS−CoV−2/human/CHN/OS3/2020:
0.000034):0.000034,SARS−CoV−2/human/CHN/OS4/2020:0.000137):0.000001,hCoV−
19/Zhejiang/HZ103/2020:0.000034):0.000034):0.000001):0.000068):0.000001,Wuhan/IPBCAMS−
WH−03/2019:0.000034):0.000001,BetaCoV/Wuhan/WH19004/2020:0.000068):
0.000001,((((SARS−CoV−2/human/CHN/Fuyang$_F$Y002/2020:0.000103,((SARS−CoV−
2/human/CHN/OS5/2020:0.000069,Brazil/SPBR−02/2020:0.000034):0.000034,SARS−
CoV−2/human/CHN/OS1/2020:0.000001):0.000001):0.000034,(SouthKorea/SNU01/2020:
0.000274,(Sweden/01/2020:0.000206,USA/CA2/2020:0.000034):0.000001):0.000001):
0.000001,Australia/VIC01/2020:0.000068):0.000034,USA/CA6/2020:0.000001):0.000001):
0.000001):0.000001):0.000001,Wuhan/IPBCAMS−WH−01/2019:0.000103):0.000001):
0.000001,2019−nCoV$_W$HU01:0.000001):0.000001,SARS−CoV−2/human/CHN/HZ−60/2020:
0.000001):0.000001,Hangzhou/HZ−1/2020:0.000001):0.000001,(Wuhan/WIV07/2019:
0.000068,(USA/CA5/2020:0.000068,USA/CA3/2020:0.000103):0.000001):0.000001):
0.000001,(((Wuhan/WIV02/2019:0.000034,BetaCoV/Wuhan/WH19008/2019:0.000001):
0.000001,USA/CA8/2020:0.000001):0.000034,Wuhan−Hu−1:0.000001):0.000001):

$0.000001):0.000001,(SARS-CoV-2/human/CHN/Wuhan_OS52/2020:0.000137,(2019-nCoV_WHU02:0.000001,(Nepal/61/2020:0.000034,SARS-CoV-2/human/CHN/Beijing-01/2020:0.000034):0.000001):0.000001):0.000001):0.000001):0.000001,(SARS-CoV-2/human/CHN/CN3/2020:0.000034,SARS-CoV-2/human/CHN/Shanghai_CH-02/2020:0.000103):0.000034):0.000068,SARS-CoV-2/human/CHN/WHUHnCoV011/2020:0.000001):0.000001,((SARS-CoV-2/human/CHN/WHUHnCoV001/2020:0.000001,SARS-CoV-2/human/CHN/WHUHnCoV004/2020:0.000001):0.000001,((SARS-CoV-2/human/CHN/WHUHnCoV020/2020:0.000001,SARS-CoV-2/human/CHN/WHUHnCoV002/2020:0.000001):0.000001,SARS-CoV-2/human/CHN/WHUHnCoV008/2020:0.000001):0.000001):0.000068):0.000001):0.000001):0.000001,((SARS-CoV-2/human/CHN/GZMU0047/2020:0.000001,SARS-CoV-2/human/CHN/OS2/2020:0.000001):0.000001,(((SARS-CoV-2/human/CHN/Beijing_IME-BJ03/2020:0.000001,((SARS-CoV-2/105/human/2020/CHN:0.000001,SARS-CoV-2/human/CHN/231/2020:0.000001):0.000001,((SARS-CoV-2/233/human/2020/CHN:0.000034,SARS-CoV-2/human/CHN/235/2020:0.000001):0.000001,SARS-CoV-2/human/CHN/Beijing_IME-BJ04/2020:0.000034):0.000001):0.000001):0.000001,SARS-CoV-2/human/CHN/HN03/2020:0.000034):0.000068,SARS-CoV-2/human/CHN/Beijing_IME-BJ07/2020:0.000171):0.000001):0.000001):0.000001,((SARS-CoV-2/human/CHN/WHUHnCoV005/2020:0.000034,(USA/IL2/2020:0.000069,USA/CA1/2020:0.000069):0.000103):0.000001,(SARS-CoV-2/human/CHN/WHUHnCoV006/2020:0.000001,((((SARS-CoV-2/human/CHN/Yunnan-01/2020:0.000001,Yunnan/IVDC-YN-003/2020:0.000001):0.000069,(SARS-CoV-2/human/CHN/WHUHnCoV007/2020:0.000034,((SARS-CoV-2/human/CHN/GZMU0044/2020:0.000001,(SARS-CoV-2/IQTC04/human/2020/CHN:0.000068,USA/CA7/2020:0.000034):0.000001):0.000001,Taiwan/NTU01/2020:0.000001):0.000001):0.000001):0.000001,SARS-CoV-2/human/CHN/GZMU0048/2020:0.000001):0.000001,((Wuhan/WH04/2020:0.000001,((Meizhou_MZ02/2020:0.000069,Meizhou_MZ01/2020:0.000034):0.000068,SARS-CoV-2/human/CHN/GZMU0016/2020:0.000001):0.000001):0.000001,SARS-CoV-2/human/CHN/WHUHnCoV012/2020:0.000001):0.000001):0.000001):0.000001):0.000001):0.000034,SARS-CoV-2/human/CHN/Shanghai_CH-03/2020:0.000068):0.000001,SARS-CoV-2/WA2/human/2020/USA:0.000103):0.000001,USA/WA1/2020:0.000001):0.000001,SARS-CoV-$

$2/human/CHN/CN4/2020:0.000137):0.007195;$

References

Baele, G., Lemey, P., and Suchard, M. A. 2016. Genealogical working distributions for bayesian model testing with phylogenetic uncertainty. *Systematic Biology*, 65(2): 250–264.

Spielman, S. J. and Wilke, C. O. 2015. Pyvolve: a flexible python module for simulating sequences along phylogenies. *PloS one*, 10(9): e0139047.
